# Infants and Their Food

**Published:** 1874-10

**Authors:** 


ARTICLE III.—"*Infants and their Food.*" *The Reviews—Summary.*

We are not disposed to extend reviews with the author of the paper above named, much less personal retorts, at the expense of the valuable space of the CHICAGO MEDICAL JOURNAL, the readers of which, and not ourselves, are the proper judges of both his paper and our review; but we cannot help wishing to add a little to that already written. We will endeavor to be brief, and therefore much that might be said, will be left unsaid. It is an axiom that writers and teachers should give their readers and pupils credit for some degree of common sense. It is not always necessary to point out in detail the connecting links between a self-evident or commonly accepted truth, and conclusions nearly related to it. Ideas are not necessarily true because of age or general acceptance, yet, having these should entitle them to a certain degree of respect, and those who would set them aside should not complain if they meet with sharp resistance. Conservatism and radicalism are ever distinctive, and at variance; sometimes one is in the right, sometimes the other. Error should be uprooted, while truth must stand. Human milk has formed the standard of infant food for about 6,000 years; and the idea exists and has always existed, of its being the only true standard. According to this idea all substitutes should come as near the standard as possible in point of actual composition and relative proportions. By the light of chemistry what is lacking in cows' milk, judged by this standard? *positively*, two elements. 1. *water;* in proportion to the whole of the solids, about twenty parts in 1,000, or two per cent.; in proportion to the nitrogenous element, the casein, and the salts, a much greater lack exists. 2. Of *sugar*, there is wanting in comparative proportion, 20 per cent. or more. There are in *excess* in cows' milk, of salts, nearly 200 per cent., *i. e.*, in the ratio of six or seven to two parts in 1000.

*Relatively* there comes in for consideration the proportion of butter; it may be deficient or in excess, according to circumstances, when used. The butter of milk not being chemically dissolved, but simply held in suspension, rapidly rises, hence the last of the milking is richer than the first, and milk poured from the top of a vessel in which it has stood but for an hour, will contain butter in excess.

Of the positive deficiencies one is a drink and the other a food, equally necessary. Does the infant thirst, milk supplies it; does it hunger, milk feeds it; if it thirst, and the standard proportion of water is lacking, it gets too much *food* in supplying its thirst, and indigestion results. If the second positive lack is not supplied, a proximate principle is deficient, and somewhere the system suffers. These are commonly accepted opinions (or more properly facts) that seem to us so plain, and so well and simply founded in common observation and common sense, as to require no long-threaded web of argument to sustain them. Are these truths? If so, did the author's paper, entitled "Infants and their Food" go with or run counter to them? We think the latter. The general object of the article, apparently, was to demonstrate if possible, first, that what we know to be lacking in the substituted food should not be added at all, because injurious when given in *excess;* secondly, of that element which might be deficient, or might be in excess, he would invariably make an addition, and that not advisedly in its own form, but in that of an agent or agents wholly foreign to the milk.

On page 141 he describes, and on the next advises the use of this "formula:" one dram of cod-liver, or oil of sweet almonds, to every three ounces of milk, being an ounce of oil to every three pints. Will the JOURNAL please allow us to restate his chief propositions? On page 132, after quoting a sensible paragraph from Wells' Chem., and flatly disputing it, he proceeds to make the following statements as a basis for further remarks:

1. "By the addition of water no dilution of casein takes place."

2. "It is not physiological to give food in a highly diluted form."

3. "The digestion and assimilation of casein is not assisted by the addition of sugar." (Who says it is, or gives it for that purpose?)

4. "It is the addition of oil that will make the artificial food a healthy substitute for the mother's milk."

In support of these propositions he writes at considerable length, and brings forward many statements by authorities, true enough in themselves, but (as we think) having no relation to such practice as he would have us follow. We have no doubt equally as much learning might be displayed in attempting to prove that black is white.

No. 1, we think we disproved fully in review, page 286; he virtually confesses its error in his review, page 462.

No. 2. Under some circumstances this is true, and others not, as the medical reader will see at a glance. Is not milk itself, with its nearly 900 parts of water, food in a highly diluted form?

No. 3, received due notice on page 287, review.

No. 4, we think has been sufficiently answered in our review, and the present paper.

These four propositions constituted the text of the author's paper, which if already proved unsound, makes it unnecessary to follow him step by step in statements and quotations mainly irrelevant.

As to whether we have or have not fully disproved his chief positions, as to whether HIS observations and inferences regarding the experiments, or *ours* were correct, is for the intelligent reader to determine.

*The review of the review.* As an exhibition of repartee this does very well, but we think he fails to touch the chief arguments of the review.

On page 462 we are accused, first, of being an old acquaintance; second, of having sore eyes; thirdly, we are quoted from with an interpolated parenthesis in this wise: "We add a *little water* (our own)" etc. Which?

Page 463 consists principally of quotations from Carpenter,

Dalton and others, going mainly to show that "drinks are immediately absorbed or otherwise disposed of," from which we are at a loss to see the proof of the author's anti-diluent theories; and further, that "a degree of solidity" (of food) is necessary to the operation of gastric juice. Does this go to show that the ordinary casein precipitate—always spongy—is too nearly solid for gastric action?

He next *admits* that he has "no objection to a little sugar, if the author pleases." That pleases us. He next asks: "Does the reviewer assert, upon his own experience, that craving sugar is a *natural* appetite?" We most assuredly do. That the appetite for saccharine substances is almost universal, strongest in children, and more particularly noticeable in cold weather, we do verily believe. "What has he to say against the assertion that children may have polyphagia and pica?" We don't know why he should assume that we are defender of morbid appetite and gluttony. The most unkind cut, however, is where water is accused of making a *cowardly retreat* from casein. Oh, what a pity water runs! But consider it *en masse*, friend. Read the concluding stanzas of "Childe Harold." Pages 464–5 are principally taken up with what authorities say about the consequence of sugar taken in *excessive* quantity, and a very wordy attempt to confuse a plain statement relating to simple redness of the mucous surface as an inflammatory indication. Then he intimates that we live in a small town, and we do think he might have spared us that; we are *so* sensitive on the point. (Not Low Point, for it is not low.)

We like his comparison as to the "knocking down," being pleased that he recognized his supineness, as further evidenced by his strenuous efforts to pick himself up.

After quoting at length from Chambers, pages 466–7, we are asked what we have to say to him ("hymn"). A good man, and we have no doubt a good singer.

We willingly join in the chorus when he sings in the praise of fat. Does the author suppose Dr. Chambers ever dreamed of composing such a dirge as "this formula of ours," page 142? Mark you, the royal food spoken of was butter and *honey!* The Hindoo laborer, whose expression is "as good ghee," is said first to devour his gallon of what? *Rice*, *starch*, *sugar*. It might have been added, that he also consumes large amounts of the product of his native *sugar cane.*

In conclusion. The author's inferential diagnosis of "calcified heart" has not disturbed our rest. The questions we have been considering are those of fact, that have an important practical bearing upon matters of life and health, and we are not so sympathetic as to accept new theories without criticism, for fear an author will not feel himself flattered. Personally, we disclaim a disposition "to pluck the credit from honest endeavor," being ever ready to accept new ideas when not inconsistent with established facts. The author ought not to presume that we have any but kindly feelings toward himself.

